# Continuous spinal anesthesia versus combined spinal epidural block for major orthopedic surgery: prospective randomized study

**DOI:** 10.1590/S1516-31802009000100003

**Published:** 2009-05-06

**Authors:** Luiz Eduardo Imbelloni, Marildo Assunção Gouveia, José Antonio Cordeiro

**Affiliations:** 1 MD, Director of Institute for Regional Anesthesia, Hospital de Base, São José do Rio Preto, São Paulo, Brazil.; 2 MD, Director of Institute for Regional Anesthesia, Hospital de Base, São José do Rio Preto, São Paulo, Brazil.; 3 PhD, Professor, Faculdade de Medicina de São José Rio Preto (FAMERP), São José do Rio Preto, São Paulo, Brazil.

**Keywords:** Anesthesia, spinal, Anesthesia, epidural, Orthopedic procedures, Bupivacaine, Anesthetics, local, Raquianestesia, Anestesia epidural, Procedimentos ortopédicos, Bupivacaína, Anestésicos locais

## Abstract

**CONTEXT AND OBJECTIVES:**

In major orthopedic surgery of the lower limbs, continuous spinal anesthesia (CSA) and combined spinal epidural anesthesia (CSE) are safe and reliable anesthesia methods. In this prospective randomized clinical study, the blockading properties and side effects of CSA were compared with single interspace CSE, among patients scheduled for major hip or knee surgery.

**DESIGN AND SETTING:**

Prospective clinical study conducted at the Institute for Regional Anesthesia, Hospital de Base, São José do Rio Preto.

**METHODS:**

240 patients scheduled for hip arthroplasty, knee arthroplasty or femoral fracture treatment were randomly assigned to receive either CSA or CSE. Blockades were performed in the lateral position at the L3-L4 interspace. Puncture success, technical difficulties, paresthesia, highest level of sensory and motor blockade, need for complementary doses of local anesthetic, degree of technical difficulties, cardiocirculatory changes and postdural puncture headache (PDPH) were recorded. At the end of the surgery, the catheter was removed and cerebrospinal fluid leakage was evaluated.

**RESULTS:**

Seven patients were excluded (three CSA and four CSE). There was significantly lower incidence of paresthesia in the CSE group. The resultant sensory blockade level was significantly higher with CSE. Complete motor blockade occurred in 110 CSA patients and in 109 CSE patients. Arterial hypotension was observed significantly more often in the CSE group. PDPH was observed in two patients of each group.

**CONCLUSION:**

Our results suggest that both CSA and CSE provided good surgical conditions with low incidence of complications. The sensory blockade level and hemodynamic changes were lower with CSA.

**CLINICAL TRIAL REGISTRATION NUMBER:**

NCT00616044

## RESUMO

**CONTEXTO E OBJETIVOS:**

Em cirurgias ortopédicas de grande porte, a raquianestesia contínua e o bloqueio combinado raqui-peridural são métodos seguros e confiáveis. Neste estudo prospectivo foram comparadas as propriedades e efeitos colaterais da raquianestesia contínua com o bloqueio combinado raqui-peridural de punção única em pacientes programados para cirurgia ortopédica de quadril, joelho e fraturas de fêmur.

**TIPO DE ESTUDO E LOCAL:**

Estudo prospectivo, conduzido no Instituto de Anestesia Regional do Hospital de Base de São José do Rio Preto.

**MÉTODOS:**

240 pacientes com cirurgias de quadril, artroplastia de joelho ou correção de fratura de fêmur programadas foram aleatoriamente arrolados para receberem raquianestesia contínua ou bloqueio combinado raqui-peridural (CSE). Os bloqueios foram realizados com o paciente na posição lateral no interespaço L3-L4. O sucesso das punções, dificuldades técnicas, parestesia, nível do bloqueio sensitivo e bloqueio motor, necessidade de doses complementares de anestésico local, grau de dificuldade técnica, alteração cardiociruculatória e cefaléia pós-punção foram registradas. Ao final da cirurgia, o cateter foi retirado e foi avaliado se havia refluxo de líquor.

**RESULTADOS:**

Sete pacientes foram excluídos (3 CSA e 4 CSE). Houve uma menor incidência significativa de parestesia no grupo CSA. O nível do bloqueio sensitivo foi significantemente mais alto no grupo CSE. Bloqueio motor completo ocorreu em 110 pacientes do grupo CSA e em 109 do grupo CSE. Hipotensão arterial foi observada significantemente mais freqüente no grupo CSE. Cefaléia pós-punção da dura-máter ocorreu em dois pacientes de cada grupo.

**CONCLUSÃO:**

Nossos resultados sugerem que ambas as técnicas provêm boa anestesia cirúrgica com baixa incidência de complicação. O nível do bloqueio sensitivo e as alterações hemodinâmicas foram menores com a raquianestesia contínua (CSA).

## INTRODUCTION

For major orthopedic surgery such as total hip or knee arthroplasty, regional anesthesia has been shown to have several advantages over general anesthesia.^1^^,^^2^^,^^3^ The most common regional techniques are spinal and epidural anesthesia, and both of these offer the advantage of having a catheter available for extending the blockade during surgery and for achieving versatile pain therapy during the postoperative period. Combined spinal epidural anesthesia (CSE) involves intentional subarachnoid blockade and epidural catheter placement during the same procedure. Continuous spinal anesthesia (CSA) is a technique for producing and maintaining spinal anesthesia with smaller doses of local anesthetic that are injected intermittently into the subarachnoid space via an indwelling catheter.^4^

In a controlled study comparing CSE, spinal anesthesia and epidural block for orthopedic surgery, it was shown that spinal anesthesia and CSE were superior to epidural block.^3^ CSA is a well-established technique that has been used successfully in many surgical procedures. It allows titration of the dose according to surgical needs and provides safe anesthesia, with minimal amounts of drugs and greater hemodynamic stability than provided by single-dose spinal anesthesia, particularly among elderly or high-risk surgical patients.^5^^,^^6^

## OBJECTIVES

In this prospective clinical study, the blockading properties and side effects of CSA were compared with single interspace CSE, among patients scheduled for major hip, femoral or knee surgery.

## METHODS

After obtaining institutional approval and informed consent from the subjects, we randomly and prospectively studied 240 ASA I-II patients (i.e. grade I-II in the classification of the American Society of Anesthesiologists) who were scheduled for femoral fracture repair or arthroplasty of either the knee or the hip. Randomization was done with the aid of a computer-generated schedule, followed by preparation of coded envelopes. The patients were assigned either to the CSA group (group 1) or to the CSE group (group 2). After one of the authors administered the anesthesia (CSA or CSE), another member of the group evaluated the protocol.

The exclusion criteria were the presence of preoperative hypovolemia; preexisting neurological disease; coagulation disorders and/or administration of thromboprophylaxis less than eight hours before the start of surgery; infection at the puncture site; agitation or delirium; or presence of a urinary bladder catheter. If accidental dural puncture were to occur during attempts to use an epidural approach with Crawford or Tuohy needles, the catheter would have to be introduced into the subarachnoid space and such patients would be excluded from the study. In the event of failure to access the epidural space within 15 minutes, single-shot spinal anesthesia would be administered and such patients would be excluded.

On arrival in the operating room, the patients were given oxygen at the rate of two liters/minute through a nasal catheter and received intravenous fentanyl 0.1 µg/kg as premedication. Standard monitoring was implemented: electrocardiography, finger pulse oximetry and non-invasive blood pressure measurement (at five-minute intervals). An intravenous preload of 100-200 ml of Ringer’s lactate solution was given over 10 minutes.

All blockades were performed in the L3-L4 interspace with the patient awake in the lateral position.

For CSA, a 22-G catheter (Spinocath^®^, B. Braun, Melsungen, Germany) over a 27-G Quincke needle was used. After identifying the epidural space with a Crawford needle, the catheter with the spinal needle inside was advanced through the epidural space until dural puncturing was felt and cerebrospinal fluid was seen in the catheter. The catheter was then fed over the needle into the intrathecal space. The spinal needle and the modified Tuohy needle were removed and a luer connector and a filter previously filled with the anesthetic solution were attached to the catheter.

CSE was performed by means of the “needle-through-needle” technique using a single interspace (Espocan^®^, B. Braun, Melsungen, Germany). The blockade consisted of performing a spinal block via a 27-G spinal needle (Spinocan^®^ 125 mm) that was introduced through an 18-G Tuohy needle (Perican^®^ 88 mm), which was oriented cranially in the epidural space. The Tuohy needle was rotated between the spinal block level and the insertion point for the epidural catheter.

With the patients still in the lateral position, plain bupivacaine 5 mg/ml was injected via the catheter in the CSA group and via the needle in the CSE group at a rate of 1 ml/15 seconds (Table 1). The level of the resulting sensory blockade was tested using pinprick tests at one-minute intervals for the first five minutes and then at five-minute intervals until reaching 15 minutes. If analgesia at level T12 was not achieved within 15 minutes, additional bupivacaine was administered through the catheter: 5 mg (1 ml) in the CSA group or 25 mg (5 ml) in the CSE group. The level of analgesia was reevaluated 15 minutes later. When the level was satisfactory, the patients were ready for surgery.

The following data were recorded: demographic data, time taken for catheter insertion, perception of dural puncturing by spinal needle, difficulty of technique (“easy”, “difficult” or “impossible”), highest level of sensory blockade, quality of motor blockade according to the Bromage scale and duration of the surgical procedure. When adequate surgical anesthesia was not achieved after 30 minutes, the technique was deemed to have failed. During surgery, the patients were given midazolam (1 mg intravenously) when complaining of discomfort or fentanyl (25 mg) when suffering from pain other than from the surgical site. At the end of the surgery, all catheters were removed and their patency was checked.

Hypotension (defined as a 30% decrease in systolic blood pressure, in comparison with preoperative control levels) was treated with ethylphenylephrine 1 mg intravenously. Bradycardia (defined as heart rate less than 50 beats/min) was treated with atropine 0.5 mg intravenously.

At the end of the surgery, all patients received 40 ml of bupivacaine 2.5 mg/ml to blockade the lumbar plexus via the psoas compartment or via the “3-in-1”-technique at the femoral nerve for postoperative analgesia.

For the first five postoperative days, patients were visited daily. Thirty days later a phone interview with regard to severe complications was held.

Student’s t-test was used to analyze of demographic data (Table 2) and other continuous variables. Mood’s test for medians, the c^2^ test and Fisher’s exact test were used when appropriate. P < 0.05 was taken to be significant. There were no sample size estimates for demonstrating particular differences, but the study power was 96% for perceiving the observed difference in paresthesia (in a one-sided setup), 90% for the observed presence of difficulty and 91% for hypotension.

**Table 1.** Dose of 0.5% isobaric bupivacaine and supplemental doses in orthopedic surgery patients

	Group 1 (CSA)	Group 2 (CSE)
150 cm - 160 cm	5.0 mg	5.0 mg
161 cm - 170 cm	7.5 mg	7.5 mg
> 170 cm	10.0 mg	10.0 mg
Supplemental dose	2.5 mg	25.0 mg

CSA = continuous spinal anesthesia; CSE = combined spinal epidural anesthesia.

**Table 2.** Orthopedic surgery patient characteristics

Variable	Group 1 (CSA)	Group 2 (CSE)
n	117	116
Gender (male/female)*	39/78	42/74
Age (years)^†^	76.1 ± 11.9	73.9 ± 10.3
Weight (kg)^†^	67.3 ± 13.7	68.7 ± 12.1
Height (cm)^†^	164.8 ± 8.7	164.2 ± 8.0

All values except sex and doses are expressed as mean ± standard deviation (SD), P > 0.005

*chi-squared test; †two-sample Student t test;

CSA = continuous spinal anesthesia; CSE = combined spinal epidural anesthesia.

## RESULTS

The patient characteristics in the two groups were comparable with regard to age, weight, height and duration of surgery. The dural puncture was successful in almost all patients. Only three patients in the CSA group and four in the CSE group had to be excluded because of unintended dural perforation with the epidural needle. The perception of dural puncturing (“click”) was the same in both groups (Table 3).

The time taken for performing the blockade was significantly shorter in the CSA group (2.6 ± 0.9 min) than in the CSE group (2.9 ± 1.2 min) (Table 3). There was greater difficulty in catheter introduction and subsequent extraction of the introducing needle in the CSE group. In the CSA group, all patients spontaneously showed cerebrospinal fluid in the catheter. In 117 patients, the catheter was inserted one or two cm into the subarachnoid space, and 20 of these patients exhibited paresthesia. Cerebrospinal fluid was obtained from 116 patients in the CSE group, and the catheter was inserted 4 to 5 cm into the epidural space. Four of these patients exhibited paresthesia, and thus there was significantly lower incidence of paresthesia in the CSE group (Table 3).

The upper limit of the sensory blockade was significantly higher when the CSE technique was used (T11 and T10) (P = 0.001). The median level in patients receiving CSA was T12 (range: T7-T12) and it was T 11 in patients receiving CSE (range: T5-T12) (Table 3).

According to the Bromage scale, the motor blockade was similar in the two groups.

In 84 patients in the CSA group and 79 in the CSE group, the first dose of 0.5% bupivacaine was sufficient to attain sensory analgesia at T12 level and thus enough for the surgical procedure. Supplemental doses were necessary in 33 CSA patients and 37 CSE patients. There were no significant differences in the supplementary doses needed in relation to time, analgesia level or blockade quality (Table 4).

Arterial hypotension was found in 17 patients in the CSE group and four patients in the CSA group, and thus it occurred significantly more often in the CSE group (P < 0.002). Bradycardia was observed in five patients from each group and postdural puncture headache (PDPH) in two patients from each group, i.e. without significant difference between the groups. There were no cases of cauda equina syndrome, transient radicular symptoms or severe complications 30 days after surgery, in either group.

**Table 3.** Spinal anesthetic characteristics in orthopedic surgery patients

Characteristics	Group 1 (CSA)	Group 2 (CSE)	P
Duration of surgery (hours)*	2.4 ± 0.8	2.4 ± 0.8	0.90
Performance time (minutes)*	2.6 ± -0.9	2.9 ± 1.2	0.007
Dural puncture^†^			
Easy/difficult	98/19	90/26	0.23
Perception of dural puncture^‡^	113	111	0.72
Catheter insertion			
Easy/difficult^†^	107/10	93/23	0.006
Paresthesia^†^	20/117	4/116	0.0005
Sensory level^‡^			0.001
T5	0	2	
T6	0	5	
T7	2	8	
T8	6	7	
T9	13	14	
T10	25	35	
T11	29	19	
T12	42	26	
Motor Blockade^‡^			0.99
3	110	109	
2	7	7	
1	0	0	
0	0	0	

*two-sample t test; †chi-squared test; ‡Mood’s test for medians.

CSA = continuous spinal anesthesia; CSE = combined spinal epidural anesthesia.

**Table 4.** Doses of bupivacaine required for the orthopedic surgeries

Doses bupivacaine	Group 1 (CSA)	Group 2 (CSE)	P
Initial dose of isobaric 0.5% bupivacaine*			
5 mg	37	27	
7.5 mg	34	32	0.987^‡^
10 mg	26	22	
> 10 mg	20	35	
Supplemental dose^†^	33/117	37/116	0.54
Level/quality	24	12	
2.5 mg	4	0	
5 mg	5	0	
7.5 mg	2	0	
10 mg	2	0	
> 10 mg	12		
Time^†^	25	25	0.97
2.5 mg	13	0	
5 mg	10	0	
7.5 mg	2	0	
10 mg	0	0	
> 10 mg	0	25	
Total anesthetic dose (mg)*	8.5 ± 3.4	18.7 ± 21.9	< 0.00005

*two-sample t test; †chi-squared test; ‡p-value from weighted least-squares method

CSA = continuous spinal anesthesia; CSE = combined spinal epidural anesthesia.

## DISCUSSION

The results from this study indicate that CSA and CSE are both effective and safe techniques for major orthopedic surgery. CSA provided better cardiovascular stability with a smaller dose of local anesthetic and shorter onset time, and without failures.

CSE was first described in the modern era for urological surgery.^7^ More recently, it has become an established technique for analgesia in labor.^8^ It is often regarded as the ideal regional technique for orthopedic surgery.^3^^,^^9^^,^^10^ CSE is the technique of choice for determining minimum intrathecal drug doses and for assessing the interaction between intrathecal and epidural drugs.

CSA was introduced in the early years of the past century.^11^ It is a well-established technique that has been used successfully in many surgical procedures.^12^ The technique allows titration of the local anesthetic dose according to surgical needs and provides safe anesthesia, particularly for elderly or high-risk patients with unstable hemodynamic status.^13^^,^^14^ The advantage of CSA in relation to CSE is that the CSA technique is easier to perform. Moreover, the intrathecal positioning of the catheter is easily confirmed by aspiration of cerebrospinal fluid.

CSA depends on how the catheter is introduced into the subarachnoid space.^15^ It is more difficult when a microcatheter is used. The Spinocath^®^ used in this study is a long catheter (72 cm), of size 22 G or 24 G, over a spinal needle of size 27 G or 29 G, with a Quincke bevel. In the CSE technique, spinal anesthesia and epidural catheter placement are performed sequentially in the patient. This has gained popularity because of the short onset time of spinal anesthesia, while the catheter provides flexibility to allow the blockade to be extended when needed.

We found technical problems during catheter insertion in 2.5% of the patients in the CSA group, which was a lower rate than found in other reports,^16^ and in 3.3% of the patients in the CSE group, which was the same as in another study.^9^

In a recent study, it was found that CSA took longer with a Spinocath^®^ 24 G (needle 29 G) than with a microcatheter, requiring 6.3 ± 3.2 min for installation.^4^ This was 2.4 times longer than what we found in our study, using the Spinocath^®^ 22 G (needle 27 G). It is well known that the time taken for cerebrospinal fluid to flow through a 29 G needle with Quincke bevel is three times longer than through a 27 G needle.^16^^,^^17^ The use of different types of needles may explain different onset times. In the CSE group, the onset time was 2.9 ± 1.2 min, the same as was published in a previous study.^10^

The CSA technique is ideal for high-risk patients in an unstable hemodynamic condition because of the possibility of injecting the local anesthetic into the subarachnoid space in incremental doses, thereby controlling the level of the sensory blockade as well as the motor blockade. Through this, greater stability is achieved for the cardiocirculatory system, with less respiratory impairment.^5^^,^^8^^,^^13^^,^^14^^,^^18^^,^^19^ CSE blockade results in a higher incidence of hypotension, occurring in 15% to 20% of the cases.^10^ The better cardiocirculatory stability observed in our CSA patients may have been due to the lesser involvement of the sympathetic system, since the highest dermatome blockaded was at least two segments lower than in the CSE patients.

Because of the incremental doses in 30% of the patients, either to produce the required analgesia or to extend analgesia, it would be useless to study the final dermatome level of analgesia. Epidural top-ups act rapidly following CSE and allow prompt evaluation of blockade level when it is too low.^20^ Subsequent doses in CSA have not been studied yet.

Comparing CSA with CSE among trauma patients, Wilhelm and Standl obtained better results with significantly smaller doses of local anesthetic and lower risk of hypotension when using CSA,^21^ while technical problems were more frequent with CSE. Those authors concluded that CSE did not have any advantage over CSA for emergency patients. In our study, we found the same degree of difficulties in both groups.

PDPH is a common complication of spinal anesthesia. The main reason for the development of a small catheter and thus a small introducing needle for it was to diminish the incidence of PDPH and thus to extend the application of the method to a wider range of patient age groups.^22^ The results from our study showed that the incidence of PDPH was 1.7%, which was in agreement with the rate of 1.6% in another study,^3^ while it was less than the rate of 3.3% for Spinocath^®^^4^ and greater than the rate of zero in two papers on the use of Espocan^®^.^7^^,^^9^

## CONCLUSION

The time taken for the blockade and cephalad dispersion of analgesia to occur and the duration of hypotension were significantly shorter in the CSA group. There was less hypotension and a lower sensory level in the CSA group.
